# Contrast-enhanced mammography-guided biopsy: principles, challenges, and opportunities

**DOI:** 10.1186/s13244-025-02148-6

**Published:** 2025-11-24

**Authors:** Rodrigo Alcantara, Javier Azcona, Mireia Pitarch, Elisenda Vall, Elisabet Vila-Trias, E. Natalia Arenas

**Affiliations:** 1 Departament de Medicine, Universitat Autònoma de Barcelona (UAB), Barcelona, Spain; 2 Radiology and Nuclear Medicine Department, Hospital del Mar, Parc de Salut Mar, Passeig Marítim de la Barceloneta, 25-29, 08003 Barcelona, Spain

**Keywords:** Biopsy, Breast neoplasms, Mammography, Contrast media, Feasibility studies

## Abstract

**Abstract:**

Contrast-enhanced mammography (CEM)-guided biopsy enables the tissue sampling of enhancing breast lesions that are not visible on conventional imaging. The technique combines dual-energy stereotactic acquisition with intravenous contrast administration, allowing accurate targeting of recombined-only lesions. It represents a practical alternative to MRI-guided biopsy, particularly in settings where MRI access is limited or contraindicated. This review examines current evidence, procedural experience, and challenges associated with CEM guidance. Early studies support its technical feasibility, although data remain scarce and heterogeneous regarding lesion selection, procedural experience, and outcome definitions. Broader implementation is challenged by equipment specifications, contrast administration practices, logistics, and reimbursement issues. As clinical adoption increases, structured patient triage pathways, standardised protocols, and prospective validation are essential. CEM-guided biopsy is a promising technique in breast imaging and has the potential to reduce reliance on MRI guidance. However, further research is required to define its role and ensure consistent performance across clinical settings.

**Critical relevance statement:**

This review critically examines current evidence, technical feasibility, and implementation challenges of contrast-enhanced mammography-guided biopsy. It highlights potential advantages for clinical settings where MRI guidance is limited, while addressing existing limitations and areas that require further research.

**Key Points:**

- Contrast-enhanced mammography-guided biopsy is a dual-energy stereotactic procedure that enables the targeting of enhancing lesions that lack conventional imaging correlates.
- The modality is accurate and feasible, though its implementation is challenged by technical heterogeneity and the absence of standardised protocols.
- Broader clinical adoption requires structured diagnostic workflows, validated contrast administration strategies, and prospective multicentre evaluation.

**Graphical Abstract:**

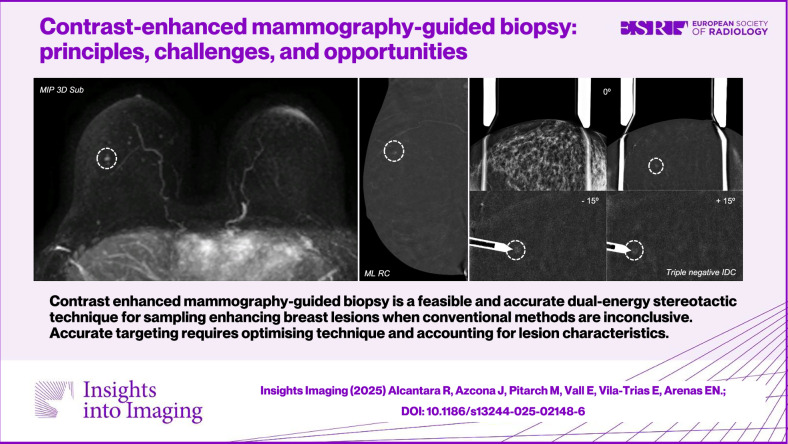

## Introduction

Contrast-enhanced mammography (CEM) is a perfusion imaging technique that, like breast MRI, uses intravenous contrast to highlight tumour neoangiogenesis [1]. It combines low-energy (LE) images, similar to standard digital mammography (DM) [2], with recombined (RC) images that depict iodine distribution, enabling visualisation of perfusion changes commonly associated with malignancy, though benign lesions may also enhance [3].

CEM is increasingly used for diagnostic problem-solving, pre-operative staging, response monitoring, and, more recently, screening in women at increased risk of breast cancer [4, 5]. However, its ability to reveal enhancing lesions not seen on conventional imaging has created new challenges for image-guided breast interventions. When lesions are visible only on RC images, without a correlate on LE, ultrasound, or digital breast tomosynthesis (DBT), MRI-guided biopsy was the only viable option until the commercialisation of CEM-guided biopsy in 2021 [6]. This method is costly, time-consuming, has limited accessibility [7], and is contraindicated in patients with non-MRI-compatible implants, severe claustrophobia, renal impairment, or gadolinium allergy [8].

CEM-guided biopsy, using dual-energy stereotactic guidance, has emerged as an alternative. Early feasibility studies suggest that it may offer a practical, accessible solution for targeting enhancing-only lesions [9, 10], with the potential to reduce reliance on MRI guidance and improve patient comfort and workflow efficiency. Nevertheless, challenges remain, including variability in lesion visibility, technical specifications, and procedural protocols.

This review summarises the current evidence, procedural experience, and practical considerations related to CEM guidance, aiming to clarify its role within the evolving landscape of image-guided breast intervention.

## Diagnostic foundations of CEM-guided biopsy

### CEM: an overview

CEM has gained widespread clinical adoption since its approval, due to its high sensitivity, moderate specificity, and compatibility with standard mammographic practice [11, 12]. While breast MRI demonstrates superior pooled diagnostic odds ratios [13], CEM offers a practical alternative for many clinical scenarios, including screening recalls and symptomatic cases [14, 15]. Meta-analyses have confirmed its strong diagnostic value and suggest that CEM may offer slightly higher specificity than MRI in some indications [16, 17].

CEM is more cost-effective [18, 19], integrates readily into existing mammographic infrastructure [20], and is often preferred by patients due to shorter examination times and greater comfort [21, 22], despite the need for breast compression. These advantages have driven its growing use, especially in centres with limited MRI access [23].

### RC images and lesion conspicuity

RC images are created by subtracting a high-energy image (HE) from LE acquisitions to highlight iodinated contrast distribution. These images form the basis for lesion targeting in CEM-guided biopsy.

According to the BI-RADS® CEM supplement [23], lesion conspicuity is defined as the degree to which an enhancing finding stands out relative to background parenchymal enhancement (BPE), and is categorised as low, moderate, or marked.

Higher BPE is often associated with dense breasts and premenopausal status [24–28]. Concordance between CEM and MRI has been demonstrated for BPE assessment [24], although MRI generally yields higher conspicuity, particularly for benign findings [29]. Lesion conspicuity likely affects biopsy feasibility, but its impact on diagnostic confidence has not yet been systematically assessed.

### RC-only findings and diagnostic implications

The term RC-only findings refers to enhancing lesions visible on RC images without a correlate on LE images, and thus not usually targetable using conventional image-guided biopsy. This terminology is consistent with current clinical practice and BI-RADS® recommendations for CEM interpretation [24].

RC-only lesions are a key indication for CEM-guided biopsy. Their reported prevalence ranges from 4% to 31.2%, with malignancy rates between 17.4% and 54.3%, influenced by differences in study design and clinical context [25–28]. A 2022 retrospective study evaluating imaging features of screening-detected abnormalities on CEM found that 50% of true-positive malignancies presented as RC-only findings [29], underscoring their clinical relevance.

The biological behaviour of RC-only findings is still under investigation, but preliminary MRI data suggest they may correspond to earlier-stage or biologically less aggressive malignancies [30]. In this context, recognising and appropriately targeting these lesions is essential in CEM-based workflows to minimise the risk of missed malignancies.

## Technical aspects and procedural considerations

### System requirements and suggested workflow

CEM-guided biopsy is a dual-energy stereotactic technique performed after intravenous administration of iodinated contrast media. It requires a mammography system capable of dual-energy acquisition, a power injector for contrast delivery, and a compatible stereotactic biopsy unit.

Currently available systems for CEM-guided biopsy are primarily upright add-ons to existing mammography units (Table 1). Pristina Serena Bright™ (GE Healthcare), Affirm® Contrast Biopsy (Hologic), and Amulet Sophinity™ (Fujifilm) support lateral and vertical needle access and can be adapted for use in the lateral decubitus position. The IMS Giotto Class S™ is currently the only system offering prone positioning for CEM-guided biopsy.

**Table 1:** Technical characteristics of CEM systems with dual-energy stereotactic biopsy capability^a^

Parameter/System	GE Healthcare Senographe Pristina^TM^	Hologic Selenia Dimensions and 3Dimensions I-View®	IMS Giotto Class^TM^ 30000	Fujifilm Amulet Sophinity^TM^
Routine LE acquisition				
Anode and filter material	Mo & Mo; Rh & Ag	W & Rh; W & Ag	W & Al	W & Rh
Filter thickness (mm)	Mo, 0.03; Ag, 0.03	0.050	0.07	0.07
Tube voltage range (kV)	26–34	25–33	30-35	26-31
Routine HE acquisition				
Anode and filter material	Mo & Cu; Rh & Cu	W & Cu	W & Cu	W & Cu
Filter thickness (mm)	0.25	0.3	0.3	0.25
Tube voltage range (kV)	49	45–49	44-49	45–49
Procedural specifications				
Biopsy setup	Add on (upright)	Add on (upright)	Add on (upright and Prone)	Add on (upright)
Regulatory approvals				
FDA 510k	Yes	Yes	Pending	Pending
CE Marked	Yes	Yes	Yes	Yes

^a^ Adapted from Jochelson MS, Lobbes MBI (2021) Contrast-enhanced mammography: state of the art. Radiology 299:36–48. 10.1148/radiol.2021201948

Intravenous contrast administration typically follows the same protocol as routine diagnostic CEM, employing iodinated contrast media at 1.5 mL/kg (up to 100–120 mL total) with a concentration of 300–350 mg I/mL, injected at 2–3 mL/s and followed by a saline flush. A two-minute delay between injection and image acquisition is critical for optimal lesion enhancement.

**Fig. 1:**
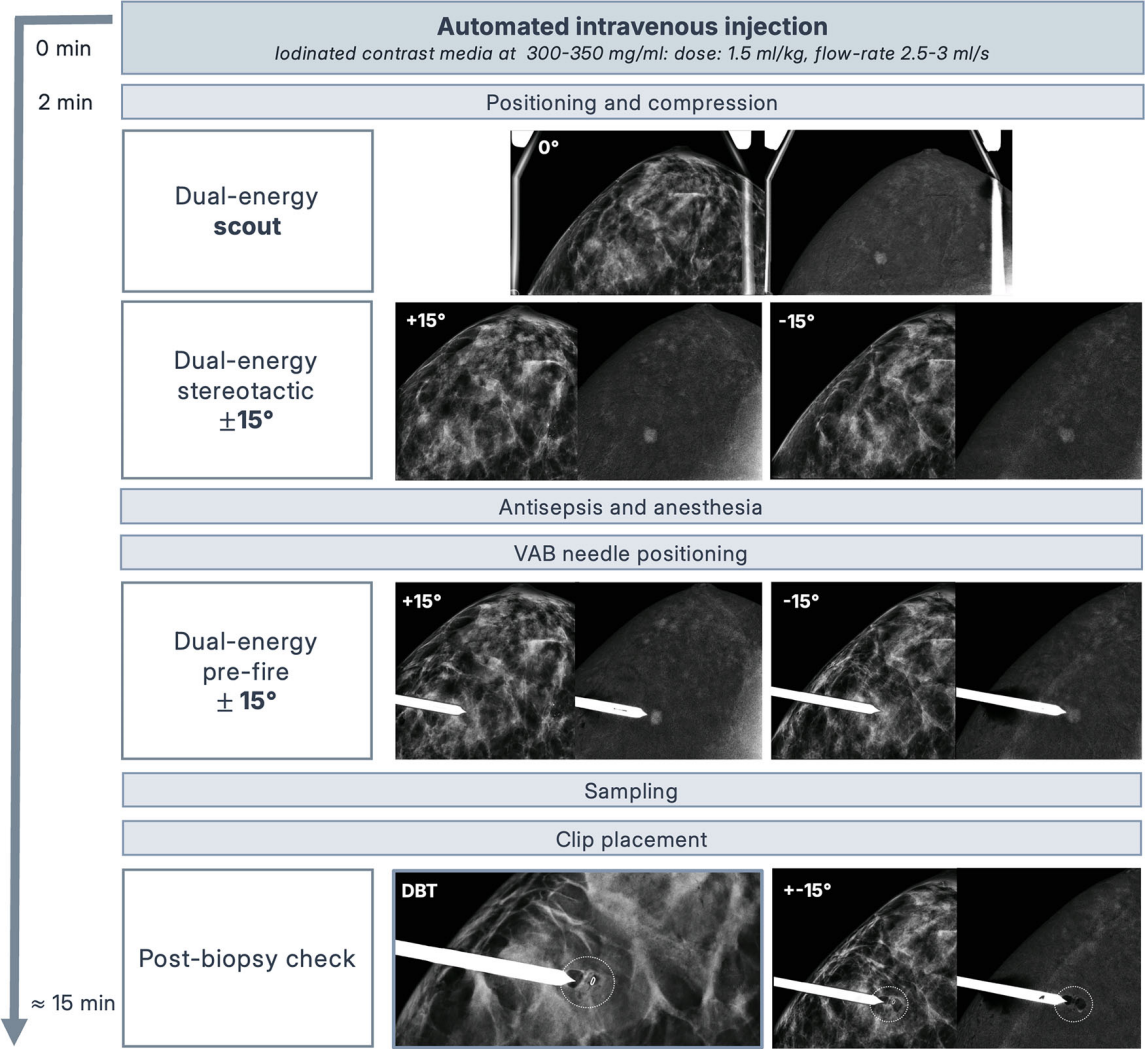
Procedural workflow of CEM-guided breast biopsy. Three-dimensional localisation of enhancing lesions is achieved using dual-energy stereotactic image pairs acquired under the same breast compression. Sequential steps include automated contrast administration, positioning and compression, stereotactic targeting (scout and ±15° pairs), vacuum-assisted biopsy (VAB) needle positioning, sampling, clip placement, and post-biopsy verification. Adapted from Alcántara et al, Eur Radiol, 2022 (CC BY 4.0 licence)

A dual-energy scout view at 0° confirms inclusion of the target in the field of view. Stereotactic angled views at ±15° are acquired for triangulation, and target coordinates are automatically calculated. Antisepsis and local anaesthesia are performed, and the biopsy needle is introduced using a fire-forward approach, if available. A pre-fire stereo pair is recommended to confirm the needle tip position before tissue sampling, which is preferably performed with a vacuum-assisted device. A clip should be placed at the biopsy site to document procedural success and enable future localisation if malignancy is identified. For RC-only targets, this step is mandatory, as these lesions may not be visible on subsequent imaging. Clip deployment is verified under compression using stereotactic or DBT views, and post-procedural mammography confirms concordance with the original finding. The procedural workflow of CEM-guided breast biopsy is summarised in Fig. 1, adapted from Alcántara et al [9].

### Patient selection and diagnostic pathway

**Fig. 2:**
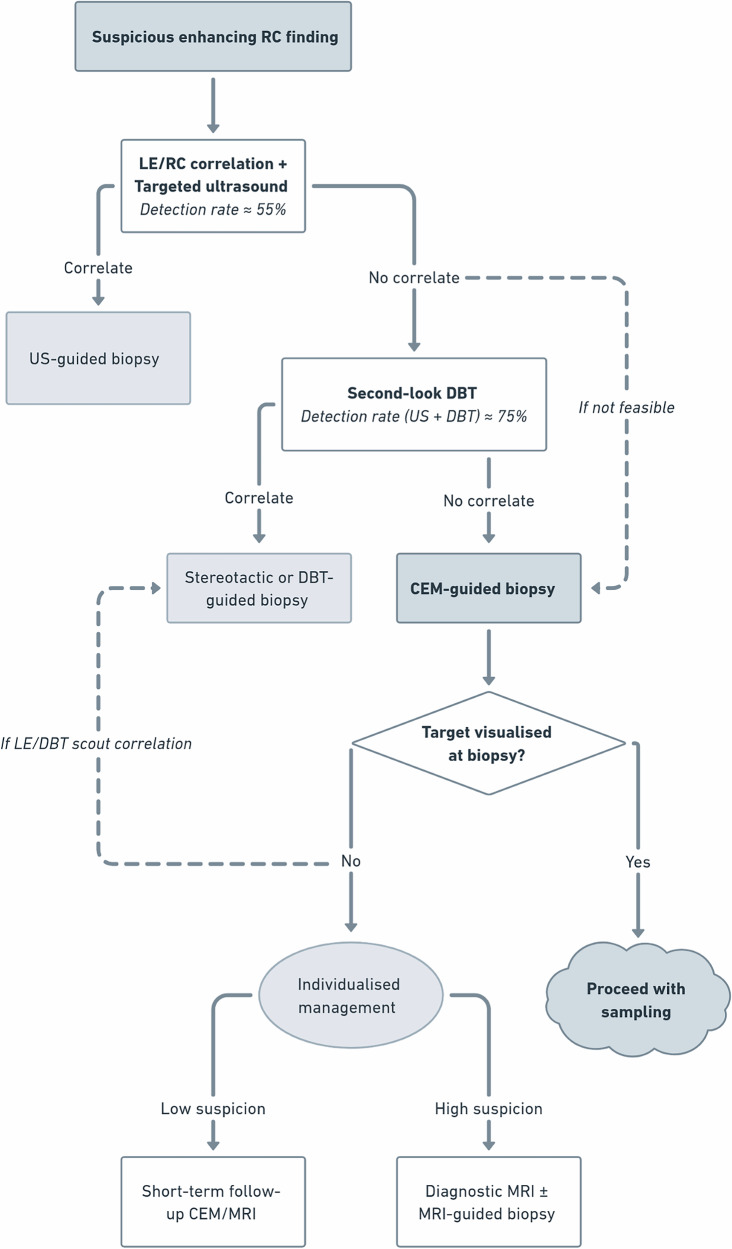
Diagnostic and biopsy pathway for suspicious RC findings on CEM. The flowchart outlines a structured approach to guide lesion correlation and biopsy selection. When a correlate is identified on ultrasound, stereotactic, or DBT imaging, the biopsy is performed under the corresponding guidance. In the absence of a correlate, CEM-guided biopsy is indicated. If the target is not visualised at biopsy, management should be individualised based on the level of suspicion and clinical context. Footnotes: Direct transition to CEM-guided biopsy may occur when second-look DBT is not available or not suitable for lesion characterisation. If a correlate or anatomical landmark is visible on LE or DBT scout images, standard stereotactic or DBT-guided targeting may be performed

When a suspicious RC finding is identified on CEM, a structured diagnostic algorithm is recommended to guide biopsy decision-making and minimise unnecessary procedures (Fig. 2). First, targeted ultrasound is performed to identify a correlate, given its accessibility and potential to downgrade [31]. LE images should be reviewed in parallel with RC images and revisited during second-look ultrasound, as they are readily available and may assist in identifying subtle morphological or anatomical landmarks.

If no correlate is found on ultrasound, either second-look DBT or direct CEM-guided biopsy may be pursued, depending on workflow and lesion characteristics. Second-look DBT can improve lesion detection in the pre-operative setting, particularly when combined with ultrasound, raising the overall detection rate from 52% to 75% [32]. In another presurgical staging study, ultrasound alone identified 55.5% of CEM-detected additional lesions, while the combined use of ultrasound and DBT increased detection to 75.8%, particularly improving characterisation of non-mass enhancement (NME) and DCIS [33]. This stepwise approach aligns with current recommendations to optimise biopsy planning [34].

In some cases, the enhancing finding may no longer be visualised at the time of the procedure, precluding sampling. If a correlate becomes apparent on the LE or DBT scout image, this can aid confirmation of the target and allow sampling to proceed accordingly [9, 35]. Otherwise, management should be individualised according to the clinical context. For low-suspicion or isolated findings, short-term follow-up CEM or MRI (according to local protocol) is advisable to confirm resolution or stability, consistent with MRI breast intervention practice parameters for non-visualised targets [36]. For high-suspicion findings, or when tissue diagnosis remains clinically necessary, such as in the setting of concurrent malignancy, further assessment with diagnostic MRI and, where appropriate, MRI-guided biopsy may be warranted [8]. Evidence to guide these decisions remains limited, reflecting the absence of standardised recommendations.

**Fig. 3:**
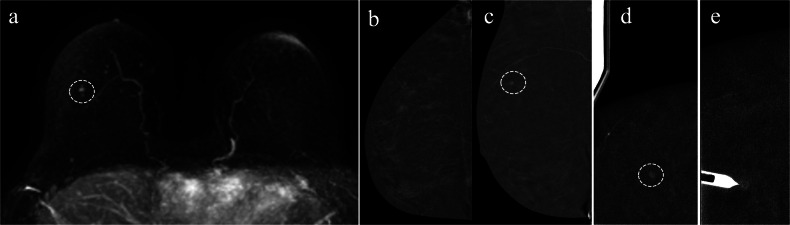
CEM in a 50-year-old woman with a history of left breast cancer and BRCA2 mutation. During follow-up, MRI identified a new 4 mm enhancing focus in the upper outer quadrant (UOQ) of the right breast (dashed circle in (**a**)), with no correlate on ultrasound. To confirm lesion visibility, a unilateral CEM was performed. Panels (**b**) and (**c**) show craniocaudal (CC) and mediolateral (ML) RC images, respectively. The lesion is more conspicuous in the upper quadrants of the ML view (dashed circle in (**c**)), which was chosen for targeting during a CEM-guided biopsy on a different day (RC scout (**d**) and pre-fire view (**e**)). HP reported high-grade triple-negative IDC

**Fig. 4:**
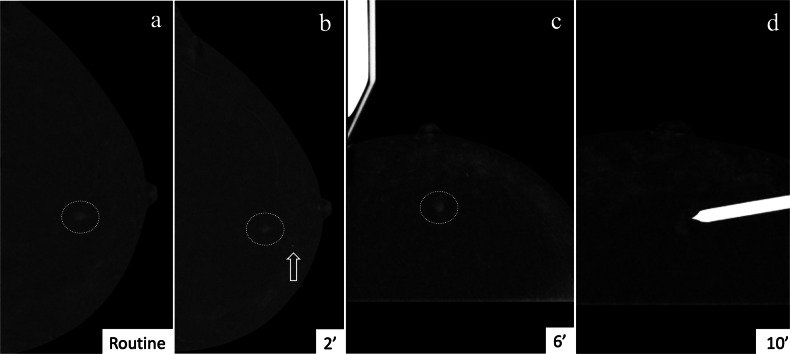
Unilateral CEM and subsequent CEM-guided biopsy with a single contrast administration. A previous ultrasound-guided biopsy of a mass enhancement seen on routine CEM (dashed circle in (**a**)) yielded a discordant histopathology result (B1). On the scheduled biopsy day, unilateral CEM was performed 2 min after contrast administration (**b**), confirming lesion persistence, and the arrow indicates the clip from the previous biopsy. The biopsy add-on was prepared, and the first scout image was acquired at 6 min (**c**), followed by the pre-fire image at 10 min (**d**). The stable lesion conspicuity allowed targeting using the same contrast bolus from the initial routine CEM. Final histopathology confirmed DCIS grade 2

For enhancing-only findings initially detected on MRI, CEM-guided biopsy may be performed directly, without prior routine CEM. This avoids duplicate contrast administration and radiation exposure. In one series, the number of scout views needed did not differ between groups who underwent routine CEM and those who did not [37]. Nonetheless, anatomical shifts between prone MRI and compressed mammography must be considered. A challenge when bypassing routine CEM is the inability to determine which view (CC, MLO, or ML) provides optimal lesion depiction, increasing the risk of targeting errors (Fig. 3). In this context, especially for less experienced users, performing at least a unilateral diagnostic CEM beforehand is advisable. A practical alternative is to acquire a unilateral diagnostic CEM on the side of the lesion before transitioning directly to the CEM-guided biopsy (Fig. 4) using the same contrast bolus. This approach minimises contrast administration to a single bolus while preserving the ability to assess the lesion’s optimal visualisation. However, one limitation of this strategy is the potential increase in BPE during the interval between diagnostic imaging and biopsy, which may reduce lesion conspicuity.

**Fig. 5:**
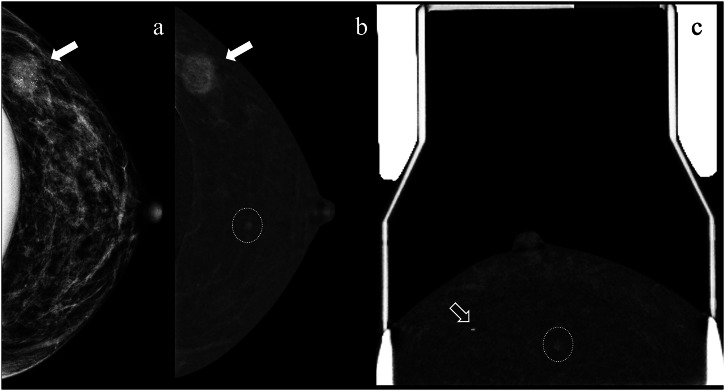
Diagnostic CEM using the implant displacement technique in a 48-year-old woman with a palpable mass in the left breast UOQ. Two enhancing lesions were identified: an irregular mass with calcifications (solid arrow in (**a**) and (**b**)), confirmed as IDC grade 3, and a millimetric enhancing mass in the central breast (dashed circle in **b**). An initial ultrasound-guided biopsy of the latter was unsuccessful. A subsequent CEM-guided biopsy (**c**) demonstrated persistent enhancement of the suspicious mass, incorrect prior clip placement (hollow arrow), and adequate implant exclusion, highlighting the feasibility of this technique in patients with implants. Histopathology revealed fibroadenomatoid changes. The patient underwent neoadjuvant chemotherapy (node-positive) and is awaiting surgery

CEM is suitable for patients with breast implants, particularly when the Eklund technique is used to displace the implant and improve lesion visualisation [38]. During biopsy, this manoeuvre also serves to shift the implant away from the needle trajectory, reducing the risk of implant damage (Fig. 5). This is particularly important when compared with MRI-guided biopsy, where improper implant positioning may increase the risk of rupture if not carefully managed [39].

### Contrast administration and timing considerations

Iodinated contrast agents approved for CT are routinely used in CEM and CEM-guided biopsy, as mammography-specific formulations remain limited. One exception is Iopromide, which received FDA approval in 2023 for mammographic use at 300 and 370 mg/mL [40].

Contrast administration should follow established guidelines and institutional protocols. Routine renal function testing is not necessary but may be considered in older or at-risk patients. According to the ESUR Guidelines [41], a ≥ 4-h interval is advised between contrast injections in patients with GFR > 30 mL/min/1.73 m², and 48 h for those with severe renal impairment. The ACR Manual [42] supports individualised risk assessment without fixed time intervals, but both emphasise minimising cumulative exposure, especially in high-risk patients.

In most cases, CEM-guided biopsy is scheduled on a separate day following the diagnostic exam. Same-day contrast administration is feasible if clinically justified. However, immediate reinjection should be avoided unless sufficient clearance is ensured, as cumulative BPE may reduce lesion visibility, as observed in our practice.

**Fig. 6:**
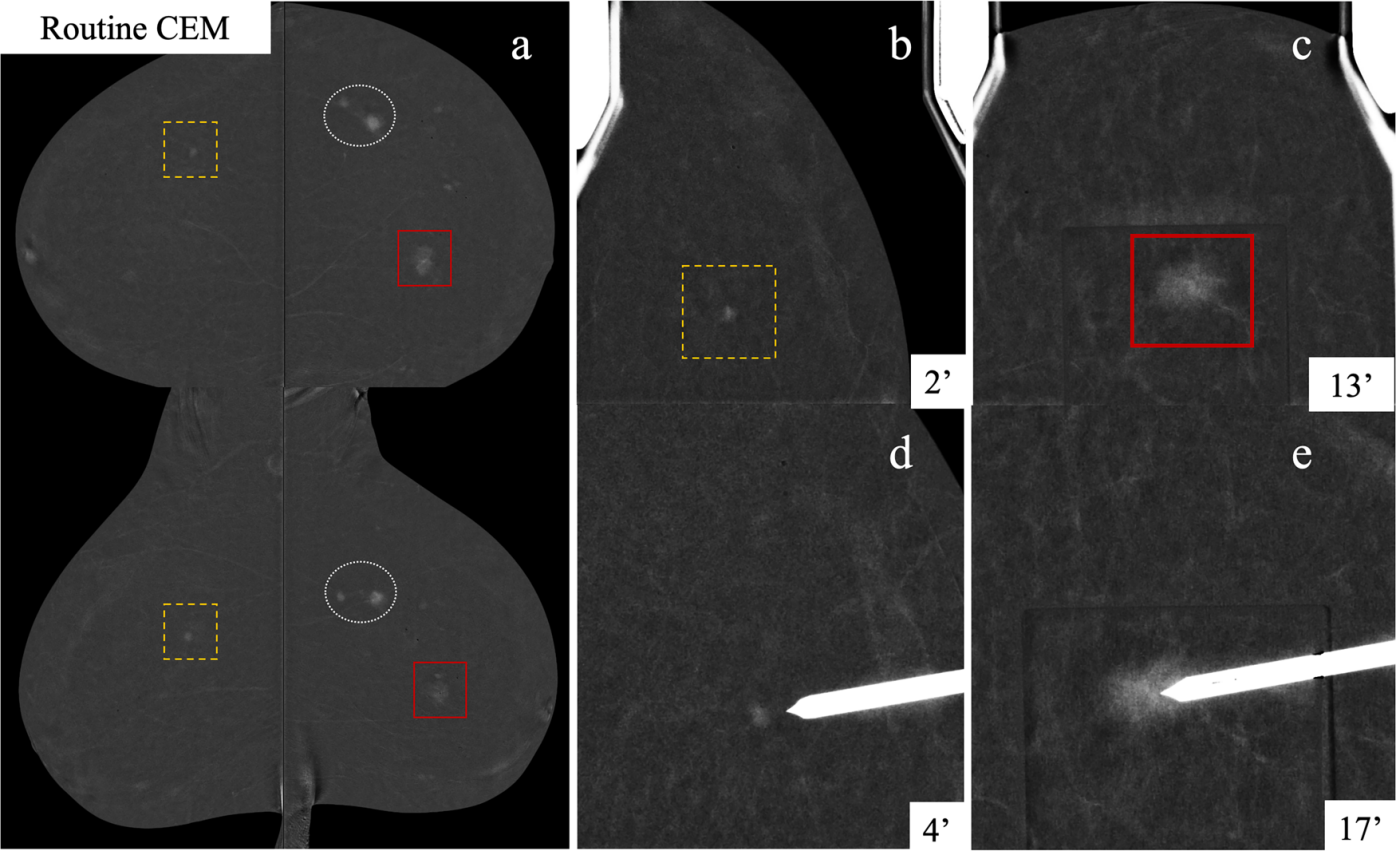
CEM in a 69-year-old woman recalled from the screening programme. Routine bilateral CEM (**a**) revealed bifocal mass enhancement (dashed white circles) in the left breast, confirmed as IDC. Additional RC-only lesions were scheduled for CEM-guided biopsy on a different day using a single contrast administration. The first procedure targeted the small mass enhancement in the right UOQ (orange dashed square in (**a**) and (**b**)), with successful targeting at 4 min (**d**). An immediate subsequent biopsy was performed on the left NME at the union of the inner quadrants (solid red square in (**a**) and (**c**)), with lesion visibility confirmed in the pre-fire image at 17 min (**e**). HP of both additional findings revealed ILC. The patient underwent a bilateral mastectomy following shared decision-making with the multidisciplinary tumour board

**Fig. 7:**
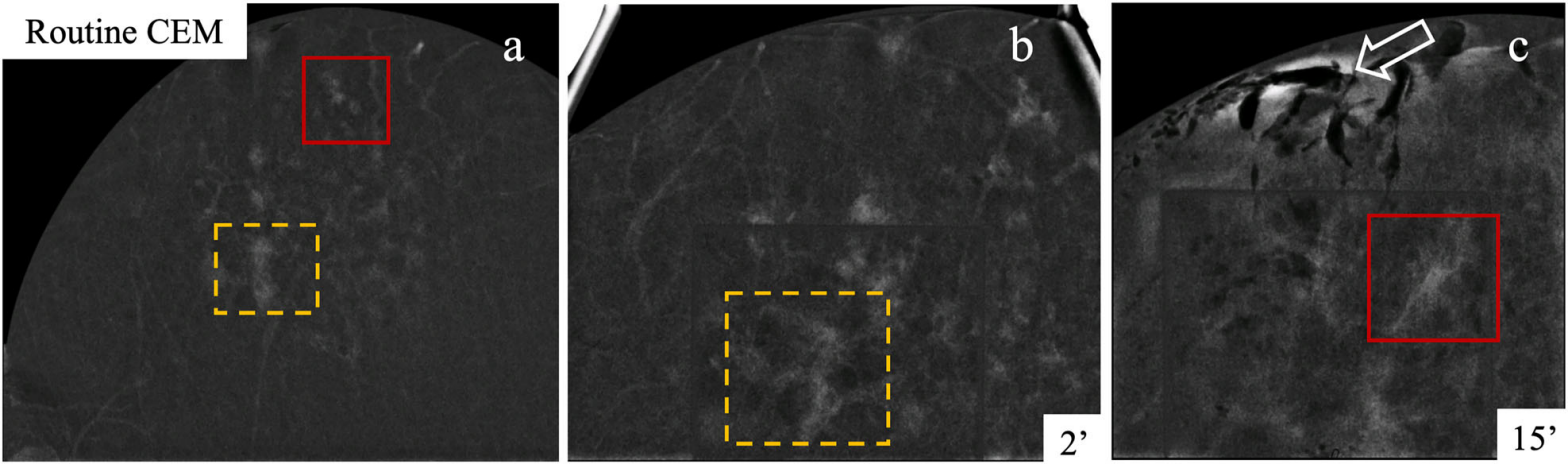
Double CEM-guided biopsy performed with a single contrast administration to assess the full extent of a clumped NME in the right breast. Routine CEM (**a**) identified two areas of interest: the posterior-central part (dashed yellow square) and the anterior (retroareolar) part (solid red square), with moderate BPE. The first biopsy targeted the posterior-central lesion at 2 min (**b**). The second biopsy, targeting the anterior lesion at 15 min (**c**), was challenged by increased BPE and artefact interposition due to air from prior anaesthesia (arrow). Both biopsies were performed under enhancement guidance. HP revealed DCIS and ADH

Enhancement may persist for over 30 min [43], enabling extended targeting and clip placement without the need for reinjection. If more than one lesion requires biopsy in the same patient, sequential sampling within the same session may be performed, provided lesion visibility and workflow permit it. If targets lie within the same field of view, biopsies can proceed without decompressing or repositioning. When lesions are in different quadrants or the contralateral breast (Fig. 6), the approach remains feasible, although provisional compression dressing at the first site is recommended to minimise bleeding. In our experience, this strategy is most effective in patients with minimal or mild BPE on prior routine CEM and requires appropriate planning (Fig. 7).

A same-day approach combining routine CEM and biopsy has also been described, involving sequential contrast injections at reduced dose per bolus (approximately 1.0 mL/kg per phase) to remain within safety limits [44]. While this may improve workflow, its effect on lesion visibility and procedural success warrants further evaluation.

Hypersensitivity reactions are rare. A systematic review of 14,012 patients identified a 0.82% pooled reaction rate for routine CEM, with most reactions classified as mild and self-limiting [45]. Similarly, interim results from the UK BRAID randomised trial recorded 24 contrast reactions among 2,035 CEM examinations (1.2%), most of which were minor, with a single severe reaction (0.05%) [46]. To date, no hypersensitivity events have been documented in published studies on CEM-guided biopsy, likely reflecting the exclusion of patients with known iodinated contrast allergies.

### Key procedural insights

CEM-guided biopsy relies on stereotactic principles familiar from conventional mammographic-guided procedures, adapted to dual-energy acquisition following intravenous contrast administration. Most commercially available platforms support seated or lateral decubitus positioning, with either vertical or lateral-arm access; prone systems remain limited to a single model.

The choice of access route and paddle configuration may influence targeting accuracy. As in conventional stereotactic biopsy, the shortest feasible needle trajectory should be prioritised, when possible, unless anatomical or technical factors dictate an alternative path.

The lateral-arm approach (parallel to the detector) is often preferred for superficial lesions or in patients with limited compressed breast thickness. In this setting, flat compression paddles are typically used, providing uniform compression and potentially improving lesion delineation [9].

The vertical approach (perpendicular to the detector), in comparison, offers greater depth along the Z-axis and is preferred for centrally located lesions or NMEs. However, this configuration often requires fenestrated paddles, which may introduce artefacts due to local variations in breast thickness and can obscure the lesion in pre-fire views due to local anaesthetic or the needle itself.

**Fig. 8:**
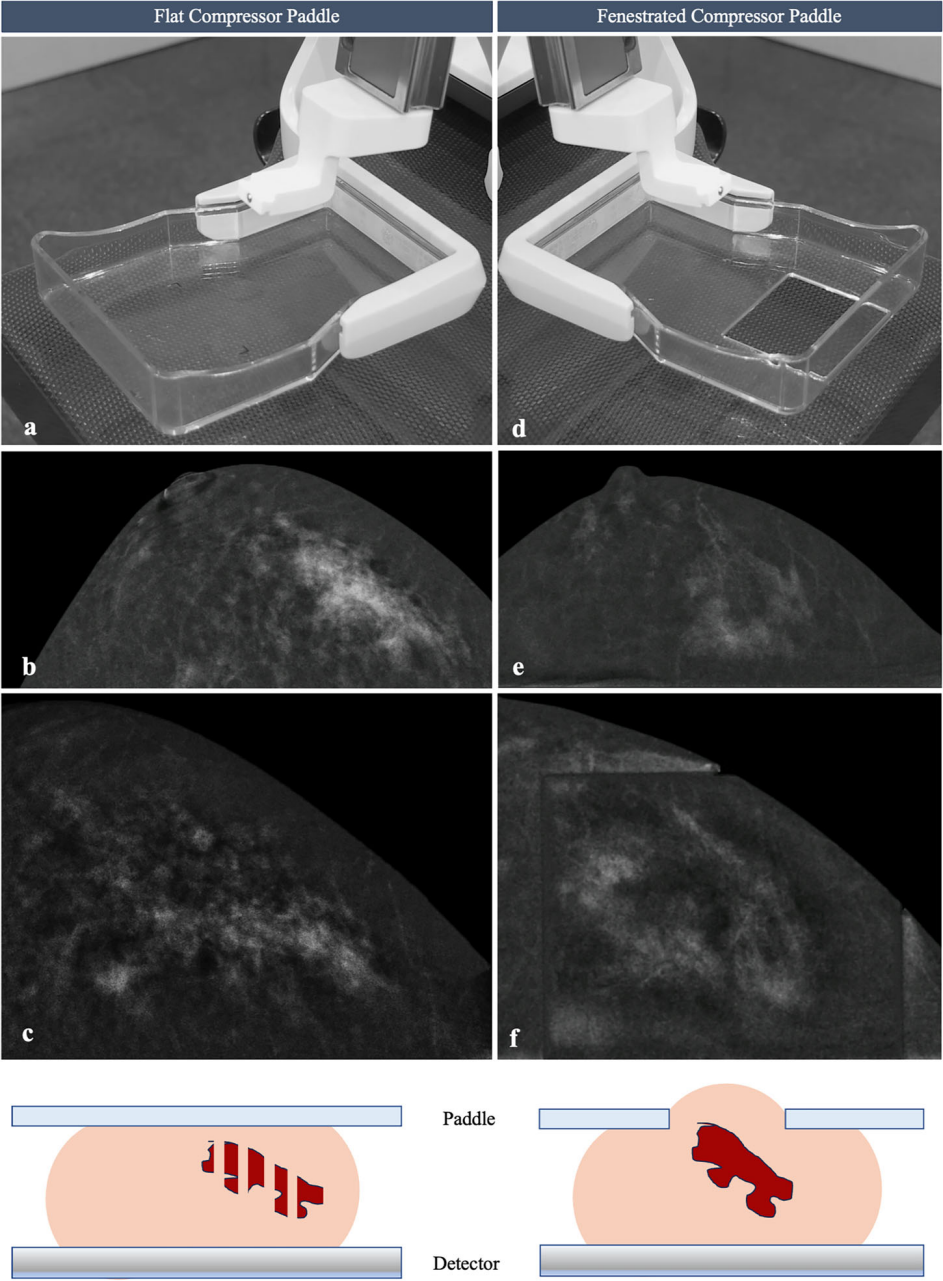
Comparison of flat (**a**) and fenestrated (**d**) compression paddles for CEM-guided biopsy of RC-only NME. On the left, a segmental homogeneous NME identified on routine CEM (**b**) shows reduced conspicuity when compressed with a flat paddle during the scout view (**c**). Histopathology (HP) revealed flat epithelial atypia and ADH. On the right, a segmental clumped NME assessed with a fenestrated paddle demonstrates consistent conspicuity between routine imaging (**e**) and procedural scout view (**f**). HP revealed multiple papillomas and ADH. In our experience, fenestrated paddles may help preserve lesion architecture and reduce fragmented appearance during compression

In lesions with subtle or diffuse enhancement, such as NMEs, excessive compression, particularly with flat paddles, may distort morphology and create a fragmented appearance. In these situations, using fenestrated paddles may help preserve lesion architecture, support visual correlation, and improve targeting consistency (Fig. 8), based on our practical experience.

### Specimen imaging and post-procedure assessment

The visualisation of iodine in CEM biopsy specimens has been proposed as a method to confirm lesion sampling. A single study found increased attenuation in low-energy radiographs of biopsy samples, interpreted as iodine retention [43]. This assumption is questioned by methodological shortcomings, such as using contrast drops in a petri dish, which are not representative of in vivo concentrations. Moreover, no histopathological or immunohistochemical confirmation of iodine presence has been provided. The observed attenuation differences in specimen images more likely reflect variations in tissue thickness or composition than true iodine content.

In current practice, immediate specimen evaluation is not performed during CEM-guided biopsy, similar to MRI guidance. Instead, post-procedure imaging is used to assess clip placement and haematoma location, ensuring sampling accuracy and excluding complications.

The optimal modality for post-procedural imaging remains undefined. Most institutions rely on standard two-view mammography or DBT. Post-procedural CEM using the same contrast bolus has demonstrated persistent enhancement in 68% of incompletely removed lesions, approximately 34 min after injection in a single-centre study [43]. However, this approach remains controversial, as post-sampling changes, haematoma formation, and increasing BPE can all reduce lesion conspicuity. Further studies are warranted to define the optimal imaging protocol and clarify the diagnostic value of post-procedural CEM.

## Evidence and clinical performance

### Feasibility and initial reported outcomes

To date, ten studies have evaluated the implementation and outcomes of CEM-guided biopsy [9, 10, 37, 43, 47–52]. While most confirm its technical feasibility, they vary considerably in design, lesion selection, and definitions of procedural success (Table 2).

**Table 2:** Overview of key studies on CEM-guided biopsy and outcomes

Study	*N* (cases)	*N* (lesions)	Main findings	Success rate	PPV3	Equipment
Alcantara et al [9]	64	66	Mass 62.1%, NME 37.9%	95.4%	39.7%	GE Healthcare
Kornecki et al [10]^a^	50	51	Focus 13.7%, Mass 21.6%, NME 64.7%	90.2%	23.9%	GE Healthcare
Kowalski et al [43]^a,b^	28	28	Mass 39%, NME 43% Other 18%	100%	25%	Hologic Inc.
Tang et al [47]	12	12	Mass 25%, NME 75%	100%	66.7%	GE Healthcare
Samarra et al (2024)	69	71	Mass 35%, NME 65%	97.1%	28%	GE Healthcare
Aribal et al [37]	28	29	Mass 7.4%, NME 92.6%	93.1%	33.3%	GE Healthcare
Layden et al [49]	15	16	Mass 6%, NME 88%, EA 6%	100%	46.7%	GE Healthcare
Nori et al [50]^b^	37	37	Mass 70.2%, NME 21.6%, EA 8.1%	100%	10.8%	IMS Giotto
Morris et al (2024)^c^	21	21	Mass 0% NME 100%	29%	0%	Hologic Inc.
Cumsky et al [52]^a,b,d^	60	63	Not reported	100%	22%	Hologic Inc.

^a^ Included lesions with correlates on LE images

^b^ Excluded lesions that did not enhance at the time of biopsy (i.e., not visible on same-day CEM or scout imaging)

^c^ CEM identified 29% of MRI-detected lesions; all lesions biopsied under CEM guidance were benign

^d^ Included 28 of 30 patients previously reported in Kowalski et al [43]

Sample sizes ranged from 12 to 69 patients, limiting generalisability. Some cohorts, such as those by Aribal and Morris et al [37, 51], included lesions initially detected on MRI rather than CEM. No data have been published on the use of the Fujifilm system for CEM-guided biopsy.

Success rates were generally high (90.2%–100%), based on accurate targeting, adequate sampling and clip confirmation on post-biopsy imaging. An exception comes from Morris et al, who found a substantially lower success rate (29%) in a series of MRI-detected lesions undergoing CEM-guided biopsy [51]. This cohort followed a same-day protocol with sequential injections using a reduced contrast dose [44] and multiple post-contrast acquisitions, which may have contributed to lesion non-visualisation.

Procedural protocol varied notably. Some studies required visible enhancement at the time of sampling [9, 37], whereas others allowed targeting based on prior imaging landmarks, including calcifications and distortions [10, 50]. Kowalski et al reported a high technical success rate (100%) based on the number of completed biopsies [43]. However, this calculation did not account for seven cases where lesions did not enhance during the biopsy session and were therefore excluded from analysis. A similar methodology was used by Cumsky et al in a recent U.S. prospective series, which also documented a 100% technical success rate based on completed biopsies [52], despite excluding 22% of enrolled patients due to lesion non-visualisation on the day of biopsy. Notably, this study partially overlapped with the earlier series by Kowalski et al, with 28 of 30 patients from one participating centre included in both cohorts. Limiting success assessment to completed procedures may overestimate real-world feasibility.

Unlike MRI-guided biopsy, which primarily targets the enhancing component, CEM-guided biopsy also enables correlation with anatomical cues on LE or DBT scout views, aiding localisation in cases of low conspicuity or fading enhancement. Interpretation of the available evidence is challenged by inconsistent definitions of lesion visibility and procedural success.

Procedural time for CEM-guided biopsy was documented in nine of the ten studies, with durations ranging from 5 to 33 min. Comparison between studies is complicated by differences in statistical reporting and measurement intervals. Some authors provided median times; others used mean values with standard deviations. The interval measured also varied, referencing either the time from contrast injection or first imaging acquisition to post-biopsy imaging.

Despite this heterogeneity, CEM guidance consistently took less time than MRI-guided biopsy. In one investigation, the mean duration of MRI-guided biopsy was 37.8 min (range 26–59), 24 min longer than the CEM-guided intervention, corresponding to a 63% reduction [37].

### Patterns in findings and histopathological outcomes

NMEs were the most frequently targeted lesion type in the available studies, accounting for 21 to 100% of cases. The proportion of enhancing masses ranged from 6% to 70%, whereas enhancing asymmetries were infrequently described, with a reported frequency ranging from 6% to 8%. Despite their diffuse morphology and associated sampling challenges, NMEs were successfully targeted in most cohorts, with technical success rates exceeding 90%. The wide variability in reported lesion types reflects inconsistencies in terminology, lesion classification, and inclusion criteria.

BPE can affect lesion conspicuity and, consequently, biopsy feasibility. Moderate or marked BPE may obscure subtle findings, particularly in angled views used during stereotactic targeting. In the series by Morris et al [51], a cancellation rate of 71% was observed for attempted CEM-guided biopsies of MRI-detected targets due to non-visualisation; baseline BPE in that cohort was mostly minimal to mild. Even so, the effect of BPE during the biopsy procedure itself, including its temporal evolution and impact on lesion visibility, was not systematically assessed and warrants further investigation. In another U.S. series [52], radiologists noted that reproducing subtle enhancement during biopsy was challenging, especially for small lesions (< 1 cm) or those seen only on a single recombined view.

Histopathological outcomes differed depending on the study population and design. Malignancy rates ranged from 10.8% to 67%, with invasive ductal carcinoma (IDC) and ductal carcinoma in situ (DCIS) being the most common malignant diagnoses. High-risk lesions such as atypical ductal hyperplasia (ADH) and lobular neoplasia accounted for 16–33.8% of cases. Benign findings included fibrocystic changes, fibroadenomas, and sclerosing adenosis.

### Breast radiation dose and image acquisition

Radiation exposure in CEM-guided biopsy exceeds that of standard CEM, due to repeated dual-energy acquisitions performed during targeting and sampling, even with collimation. The mean average glandular dose (AGD) for a bilateral two-view full-field CEM is approximately 4.9 mGy [53]. In comparison, CEM-guided procedures generally involve at least 6–10 acquisitions, with a recent publication estimating a mean AGD of 11.8 mGy [54]. Higher values, such as 20.6 mGy, have also been reported in smaller cohorts [49], likely reflecting variations in operator experience and procedural efficiency.

Evidence on mean AGD across the three mammographic biopsy techniques remains limited to a single study conducted using a vendor-specific platform [54]. In that analysis, CEM-guided and conventional stereotactic biopsy showed nearly identical mean AGD per acquisition (~1.48–1.49 mGy) and per procedure (~11.8–11.9 mGy). DBT-guided biopsy had slightly higher mean AGD per acquisition but a lower total mean AGD per procedure (~6.2 mGy), reflecting fewer required acquisitions. Nonetheless, efforts should continue to minimise cumulative exposure, particularly in patients requiring multiple procedures, and further research across platforms is needed to inform standardised optimisation strategies.

## Implementation challenges and opportunities

### System availability and workflow integration

Despite supporting evidence from feasibility studies, the implementation of CEM-guided biopsy in routine clinical practice faces technical, logistical, and workflow-related barriers. Beyond baseline infrastructure, broader adoption requires biopsy guidance software, staff training, and procedural integration. A compatible contrast injector, typically the same device used for routine CEM, is also necessary.

Currently, only a few commercial platforms support CEM-guided biopsy, primarily as upright add-on units. A prone system with this capability (Giotto Class Biopsy CEM, IMS Giotto, Bologna, Italy) has recently become available; it holds CE certification and is commercialised in markets where the platform is registered, except in the USA, Canada, Australia, and Japan.

Practical setup considerations include optimising patient positioning, ensuring efficient lesion targeting under time constraints, and integrating contrast-based workflows into existing biopsy protocols. Real-time localisation can be demanding, particularly for faint or transient enhancement, and scout images in LE or DBT mode should be considered in challenging cases [35].

Workflow logistics add another layer of complexity. While same-day biopsy may be feasible in some institutions, it depends on streamlined coordination between radiologists, technologists, and scheduling teams. In most cases, biopsies are scheduled separately, with strict adherence to contrast protocols to ensure safety and optimal lesion visibility.

From our institutional experience, a few practical measures have proven useful during early implementation. Careful pre-procedural review of each case by the biopsy team facilitates optimal positioning strategy, anticipates breast thickness limitations, and helps determine the appropriate approach or need for breast spacers. Allocating extended slots during the initial adoption phase allows for familiarity with the workflow and image review timing. For training purposes, selecting cases with unequivocal enhancement facilitates learning without added complexity.

To date, no formal learning curve has been established for CEM-guided biopsy. A single study has reported a learning effect in routine diagnostic CEM, with improved diagnostic performance after interpretation of approximately 75 cases [55]. In one series, the authors noted that after the first seven biopsy cases, they omitted prior diagnostic CEM and proceeded directly to biopsy using CEM scout views for MRI-detected lesions [37], reflecting a workflow adjustment over time.

### Variability in imaging parameters and equipment

Standardising RC-image across both routine and interventional CEM is challenged by differences in imaging parameters, detector technology, and post-processing algorithms between vendors. Technical factors such as automatic exposure control (AEC) and dual-energy subtraction methods can affect image quality and iodine conspicuity [56, 57]. These variations may influence lesion depiction and targeting feasibility, although their direct clinical impact remains underexplored. Further studies are warranted to clarify the interplay between equipment characteristics, operator expertise, and procedural outcomes.

In one publication, a low technical success rate was observed using a specific vendor system [51]. In comparison, other centres using the same equipment reported substantially higher success rates [43, 52], suggesting that reader experience, lesion selection, or workflow differences may also contribute [58].

### Patient comfort and procedure-related events

CEM-guided biopsy has shown favourable tolerability in feasibility studies. No major interruptions or dropouts due to discomfort were reported, and vasovagal reactions occurred in 3.2% of cases in upright settings [9]. In one cohort, half of the patients experienced no pain, and the remainder described mild to moderate symptoms [49]. Similar findings were observed in a separate investigation, where patient satisfaction surveys indicated overall positive experiences, although lower scores were noted for comfort and perceived duration [43]. The two main areas identified for improvement were adequate local anaesthesia and better pre-procedural information.

Haematoma formation is the most frequently documented complication. Mild haematomas were observed in approximately 25% of patients and were self-limited [9, 50]. A single case of severe haematoma managed surgically was described [50], but no cases of delayed complication or rebleeding were reported. CEM-guided and conventional stereotactic biopsies are technically similar, and both commonly use vacuum-assisted devices. However, CEM guidance is often applied to lesions not visible on standard imaging, and, as in MRI-guided biopsy, larger sampling is frequently pursued to ensure adequate histopathological representation [59]. This may increase the likelihood of haematoma formation. Optimising local anaesthetic technique may enhance patient comfort, whereas incorporating vasoconstrictors can help reduce bleeding risks [60]. Post-biopsy compression and brief observation remain essential.

### Future directions and research priorities

Despite promising technical and clinical results, both CEM and CEM-guided biopsy are not yet widely implemented. Limited equipment availability, the need for additional software licences, and inconsistencies in reimbursement policies represent major obstacles [61].

A recent international survey confirmed substantial variability in reimbursement for routine CEM, with few centres having access to dedicated billing codes and many relying on alternative strategies [62]. Moreover, no specific procedural codes currently exist for CEM-guided biopsy, which may discourage investment in equipment or staff training. In this context, recent prospective U.S. data suggest that CEM-guided biopsy offers shorter procedure times and approximately 45% lower cost compared to MRI-guided biopsy, supporting its operational value [52]. It should be noted that this analysis was based on per-procedure reimbursement estimates and did not incorporate non-visualised or cancelled cases, which were documented descriptively in the same study.

Standardisation of routine and interventional protocols is relevant to achieving reproducibility in lesion targeting and post-biopsy evaluation. Technological advancements, including a more robust RC algorithm and improved artifact reduction, could help optimise procedural accuracy.

The influence of lesion characteristics, such as size, enhancement pattern, location, and background enhancement, on biopsy performance has yet to be fully investigated. In parallel, efforts to optimise patient-centred strategies should be central to ongoing developments in image-guided breast interventions.

## Conclusion

CEM-guided biopsy is an emerging image-guided technique that enables tissue sampling of enhancing lesions not visible on conventional mammography or ultrasound, potentially serving as an alternative when MRI-guided biopsy is not feasible. Early investigations have demonstrated technical feasibility, but available evidence is preliminary and marked by variation in lesion selection, workflow protocols, and outcome definitions.

Successful implementation will depend on thoughtful evaluation of system availability, procedural logistics, and coherent contrast protocols. Factors including lesion morphology, biopsy timing, and BPE may influence lesion conspicuity and targeting accuracy. Differences in equipment platforms and proprietary image reconstruction algorithms also potentially affect lesion visibility and procedural outcomes.

As clinical use expands, continued research is needed to support patient selection strategies and ensure consistent performance across settings.

## Abbreviations

AGD: Average glandular dose
CEM: Contrast-enhanced mammography
DBT: Digital breast tomosynthesis
HE: High-energy image
LE: Low-energy image
